# Humanized childbirth in Colombia: Prevalence and associated factors

**DOI:** 10.1371/journal.pone.0326766

**Published:** 2025-07-11

**Authors:** Myriam Ruiz-Rodríguez, Arlin Martha Bibiana Pérez-Hernández, Flor de María Cáceres-Manrique, Cristina María Mejía-Merino, Elba María De La Cruz Bermúdez-Quintana, Darlynne Xiomara Alfaro-Benavides, Adrián Arcenio Hidalgo-Erazo, Fabio Alberto Camargo-Figuera

**Affiliations:** 1 Medicine School-Department of Public Health, Universidad Industrial de Santander, Bucaramanga, Colombia; 2 School of Medicine, Universidad El Bosque, Bogotá, Colombia; 3 Faculty of Health Sciences -Department of Public Health and Epidemiology, Pontificia Universidad Javeriana de Cali, Cali, Colombia; 4 Faculty of Health Sciences – Universidad de Nariño, Pasto, Colombia; 5 Nursing School, Universidad Industrial de Santander, Bucaramanga, Colombia; Fundacao Oswaldo Cruz Instituto Rene Rachou, BRAZIL

## Abstract

In several parts of the world, women are subjected to disrespectful and offensive treatment during childbirth. Humanized obstetrics or humanized childbirth has emerged as a response to this situation, and it refers to a model of comprehensive care for labor and childbirth that focuses on the respect and dignity of the woman. This study was aimed at estimating the prevalence and factors associated with humanized childbirth, as perceived by women in six major cities in Colombia. An analytical, multicenter, representative cross-sectional study was conducted in a random sample of six Colombian cities, including the country’s capital. We included 1294 women who were over 14 years of age and who had an institution-assisted vaginal childbirth resulting in a live birth in 2018. The prevalence of humanized childbirth in the sample of participating cities was 32.9% (95% confidence interval, 30.4–35.5). The city of residence of the pregnant woman, the presence of health problems during pregnancy, attendance at an antenatal course, the sex of the attending professional, and the association between the involvement of an obstetrician–gynecologist and the occurrence of a humanized childbirth were examined. These results indicate the need to monitor compliance with existing regulations to ensure dignified, respectful, and humane care that prioritizes women.

## Introduction

Childbirth is a transformative event in a woman’s life, ideally a positive and safe experience free of complications, where both mother and baby receive appropriate care. However, evidence indicates that many pregnant women globally face disrespectful and offensive treatment during childbirth, violating their rights and endangering their health [1,2]. To counter this, the concept of humanized maternity care has emerged, emphasizing respect for women’s dignity throughout pregnancy, labor, delivery, and postpartum. It adapts to each woman’s sociocultural context and beliefs, promoting physical and emotional well-being for mother and newborn while reducing maternal and perinatal morbidity and mortality [3–8].

Humanized childbirth involves practices such as allowing women to have a trusted companion, avoiding unnecessary interventions, ensuring participation in decision-making, and fostering a calm and respectful environment. This care reduces stress and anxiety, shortens recovery and hospital stays, and facilitates better outcomes, including stronger maternal–child bonding and early breastfeeding through immediate skin-to-skin contact [9]. Similarly, respectful maternal care emphasizes human rights protection, emotional support, and the elimination of coercive practices, underscoring the importance of dignity in maternal health care [9]. Both humanized childbirth and respectful maternal care are essential strategies for maternal health, focusing on the care of women but with different priorities.

In recent years, global efforts to promote respectful and high-quality maternal care have gained momentum. The World Health Organization (WHO) declared in 2014 that all women have the right to dignified care during childbirth, free from violence and discrimination [2]. However, progress has been uneven. In Latin America, few countries have implemented data collection systems or enacted legislation to support these principles [10]. Colombia has made strides with the passage of Law 2244 in 2022, which guarantees women’s autonomy, dignified treatment, and access to quality health services during pregnancy and childbirth. This legislation aims to uphold women’s rights and improve maternal care [11].

Despite these advances, adherence to WHO recommendations for humanized care remains low in Latin America [8,12–16]. In Colombia, most studies on humanized childbirth are qualitative and focus on women’s perceptions of maternity care in specific cities [17,18]; evidence suggests slow implementation of WHO guidelines [19], and there are no quantitative studies on the prevalence of humanized childbirth or the factors influencing its practice. This gap limits the ability to assess guideline adherence, inform health policies, and evaluate the impact of Colombia’s Humanized Birth Law.

To address this, the present study aims to estimate the prevalence of humanized childbirth and identify associated factors from the perspective of women in Colombia’s six main cities: Bogotá, Medellín, Cali, Bucaramanga, Manizales, and Pasto. This research will provide critical evidence to guide decision-making, promote the humanization of care, and evaluate the success of policies such as Law 2244.

## Materials and methods

### Study design

A cross-sectional, analytical study was conducted in six major cities in Colombia, including the country’s capital. The analysis presented in this article is part of a broader study that examined the prevalence of humanized childbirth among women from urban areas in six Colombian cities who had a live birth (either vaginal or cesarean).

### Context

This study was conducted between September 2020 and April 2021 in Colombia, a middle-income country divided into 32 departments, 4 special districts, and 1 capital district (Bogotá). According to updates from the most recent 2018 census, Colombia has a population of 49,500,000 million [20]. The national maternal/mortality ratio (MMR) for 2021 was 83.2 for 100.000 live births [21]. Six cities were included in this study: Bogotá (center of the country), Bucaramanga (northeast of the country), Manizales (center-west of the country), Medellín (northwest of the country), Cali, and Pasto (southwest of the country). In 2021, 616,914 live births occurred in these six cities, representing 25% of the country’s total births [22]. These cities have prenatal care and institutional childbirth coverage of more than 90%, and MMR values are lower than the national MMR.

Colombia has implemented a social security health system since 1993. One is contributory, for those with formal employment and the ability to pay; another is subsidized, for the population without the ability to pay; and a third is the special regime, which provides coverage for workers and their families belonging to sectors such as the hydrocarbon industry, teachers, and the military. Currently, more than 95% of the country’s population is covered.

Although care during pregnancy, childbirth, and the postpartum period is universal and covered by health insurance, the conditions of access, quality, and equity vary from city to city. This is because the organization of the care network is defined and controlled by the insurance companies through service contracts between insurers and public or private health care providers. In each city, there is a local health authority (Municipal Health Secretariat) that is responsible for supervising insurance and the provision of health services.

### Sample

For the calculation of the sample size [23], simple random probabilistic sampling was used with the following parameters. The expected prevalence of humanized delivery is 50%, the reference population of live births in each city is based on the vital statistics published by the National Administration of Statistics (DANE) for 2016 [22], a statistical power of 80%, a confidence level of 95%, an accepted margin of error of 7% and a variance correction factor for the design effect of 1.2, given the sampling strategy implemented by city, giving a total sample size of women having a vaginal delivery of 1294.

### Participants

Women were included if they met the following criteria: they were older than 14 years, lived in urban areas of the six selected cities, had an institutional childbirth with a live birth in 2018, the newborn was still alive at the time of the survey, and they had no problem answering the questionnaire. No exclusion criteria were considered.

All study participants formalized their participation by signing an informed consent form. In the specific case of minors, in addition to their informed consent, their parents or guardians provided authorization by signing an assent form after being informed of the characteristics and objectives of the study.

Simple random probability sampling with replacement was used as the sampling frame independently in each city, using the official list of the Registro Único de Afiliados (Unique Affiliate Registration), which corresponds to the list of births attended in each city in 2018. The Registro Único de Afiliados contains information on pregnancies and births of women affiliated with and not affiliated with the health system. Of the potential participants, 142 (9.5%) could not be reached, and 54 (3.6%) declined to participate

### Data collection instrument

The research team developed and validated the “humanized childbirth questionnaire” (HDQ), which includes questions that aim to capture, in the best way possible in the Colombian context, the respondents’ perceptions of humanized care at each stage of their care and in the internationally recognized areas of respectful childbirth.

According to their nature, responses to the items of the instrument were dichotomous (yes/no) in some cases and on a Likert-type scale from 1 to 3, where 1 corresponded to the fact that the aspect addressed by the question did not meet the criterion of humanization of care, 2 to low compliance, and 3 to full compliance. For face validity, the properties of clarity and coherence were assessed. Conversely, for content validity, the pertinence and relevance of each question were evaluated by a panel of 31 experts. These experts comprised 24 national and 7 international members, all with experience in research, teaching, or assistance in the field of obstetric care. Among them were 11 medical professionals (4 specialists and 7 subspecialists), 6 nurses (1 nurse without additional studies, 3 with specialization, and 2 with a master’s degree), 2 psychologists, and 1 sociologist. The instrument obtained a face validity index of 0.89 and a content validity index of 0.91. These results are available in a previous publication [24]. In addition, the construct validity of the instrument was assessed, and the results are presented below.

Additionally, between December 2019 and January 2020, a pilot test of the information collection process was conducted with 40 women in each city who had given birth in 2018 and agreed to be interviewed. The interviewers recorded in a field diary the relevant aspects about the understanding of the questions, the time of the interview, the logistical aspects of the survey application, and any other striking findings about the questions asked. The research team, together with the interviewers, analyzed the field diaries, identified relevant issues, and adjusted the form and process.

The instrument consisted of 74 items, organized in the same consecutive order as the stages of maternal care, as follows: 1. Prenatal care; 2. Admission and preparation for delivery; 3. Labor; 4. Delivery; and 5. Postpartum (S1 Appendix).

### Data collection

Data collection was conducted through the application of the HDQ via a telephone interview. The information was collected between September 2020 and April 2021 and conducted by interviewers with an undergraduate degree in health or social sciences. The interviewers were trained through a specially designed training that was jointly developed and standardized for all cities.

Once the process was standardized and the tool for identifying humanized births was validated, the information was collected using the Primary Health Care data collection and a management platform called GESTOR-APS, a platform used by the Bucaramanga Health Department and the Department of Santander to manage Primary Health Care program. To ensure the correct use of this platform, the surveyors were trained and a pilot test of data collection and management was conducted.

### Construction of the humanized childbirth index

There is still no consensus in the scientific community on how to measure the construct of humanized childbirth nor is there a questionnaire that is considered the “gold standard.” The experience of childbirth is a vital, multidimensional event that involves different stages of care, including clinical, physiological, psychological, organizational, and subjective processes and is influenced by the environmental context [25]. To best capture this complexity and because different stages of maternal care were assessed, this study constructed a synthetic index to measure the woman’s experience of the humanized care of vaginal childbirth.

The construction of this index included the following phases [26–30]: a) normalization or standardization; b) calculation of construct validity; c) calculation of partial Ip indices [28]; and d) calculation of the synthetic index of the woman’s experience with humanized childbirth (ISMPH, by its acronym in Spanish). A detailed description of the methodology is provided in S2 Appendix. The ISMPH ranges from 0 to 100, where 0 represents the lowest score for delivery care and 100 represents the best. Scores of ≥85 were considered to reflect the best experience of humanized childbirth care, which is the primary outcome of this study and will henceforth will be referred to as humanized childbirth.

### Data analysis

A descriptive analysis of the demographic and gestational characteristics of the study participants (n = 1294) was conducted. The prevalence of humanized birth was calculated with 95% confidence interval (95% CI) for the total sample and stratified for each variable. Crude prevalence ratios (PRs) with their 95% CIs were calculated using Poisson regression to evaluate the associated factors. To obtain adjusted associations, Poisson regression with robust variance was used, considering humanized childbirth as the outcome.

A strategy based on the “four-level hierarchical conceptual model” was used to construct a multivariate model [31], in which sociodemographic variables were included at a more distal first level: city, marital status, and maternal age at childbirth. At the second level, socioeconomic variables were included: schooling, socioeconomic class, work, health system affiliation, home ownership, and vulnerability conditions. At the third level, variables related to pregnancy were included: pregnancy planning, number of living children, health problems during pregnancy, high-risk obstetric pregnancy, and attendance at antenatal and postnatal classes. Finally, at the fourth level, the most proximal variables related to the childbirth care were included: time of labor, type of health facility, and type and sex of the professional who performed the childbirth care. In addition, an assessment of multicollinearity between all the potentially associated factors was performed using a Spearman correlation matrix.

For the multivariate analysis, a backward selection strategy was used. Initially, all variables from the same hierarchical level were included, and those with p > 0.20 were subsequently excluded. The variables from the next level were adjusted for all variables from the same level, as well as those from the previous level that remained in the model.This process was repeated for the other levels. Factors with p < 0.05 were considered to be associated with humanized childbirth. In the multivariate analysis, the PRs were reported with their 95% CIs and the p-value of the variable obtained by the Wald test of the Poisson regression model with robust variance. Finally, goodness-of-fit, deviance, chi-squared, and model specification tests were performed. All statistical analyses were carried out in Stata (version 15.1).

### Ethical aspects

This research is considered minimal risk research because it is an observational study. Study participation was formalized by signing an informed consent form. The study protocol was approved by the Institutional Research Ethics Committee of the Universidad El Bosque, in the extraordinary meeting of May 10, 2018, Act N° 012–2018.

## Results

A total of 1294 women who gave birth vaginally were interviewed in 2018 in the six cities that made up the sample. We found that 75.4% were living with a partner at the time of childbirth, 81.6% had a secondary education or higher, 68% belonged to a low socioeconomic level, and 63% lived in rented housing. Regarding the characteristics of the pregnancy, 52.4% of the women did not plan their pregnancy; 32.9% had some health problem during pregnancy; 47.3% of the women had a pregnancy classified as risky; 68.1% had a duration of labor of less than 11 hours; 26.2% of the women did not know if the professional attending the childbirth was a general practitioner, and 20% of them did not know if the professional attending the childbirth was a specialist in gynecology and obstetrics (Table 1).

**Table 1:** Prevalence of humanized delivery according to sociodemographic and gestational characteristics.

	Frequency n = 1294		Humanized Childbirth n = 1294	
Variable	n	%	Prevalence	95% CI
Total prevalence of humanized childbirth			32.9	30.4–35.5
City				
Bogotá	147	11.4	21.7	15.8–29.1
Bucaramanga	209	16.2	10.0	6.6–14.9
Cali	203	15.7	43.8	37.1–50.7
Manizales	272	21.0	41.9	36.1–47.8
Medellín	324	25.0	41.6	36.4–47.1
Pasto	139	10.7	25.1	18.6–33.0
Marital status at childbirth				
No partner	319	24.6	32.6	27.6–37.9
With partner	975	75.4	33.0	30.1–36.0
Age at childbirth				
14–17 years old	108	8.4	30.5	22.6–39.8
18–26 years old	645	49.9	32.5	29.0–36.2
27–34 years old	409	31.7	33.9	29.5–38.7
≥35 years	130	10.0	33.8	26.2–42.3
Education				
Elementary	238	18.4	34.4	28.6–40.7
Secondary	751	58.0	32.4	29.2–35.9
Higher education	305	23.6	32.7	27.7–38.2
Socioeconomic stratum				
Under	880	68.0	33.8	30.8–37.9
Medium	393	30.4	30.2	25.9–35.0
High	21	1.6	42.8	23.9–64.0
Labor available at the time of childbirth				
No	742	57.4	33.1	29.8–36.6
Yes	551	42.6	32.6	28.8–36.7
Affiliation with the general health social security system				
Contributory	681	52.6	34.2	30.7–37.8
Subsidized	556	43.0	32.5	28.7–36.5
Special	21	1.6	28.5	13.4–50.7
Does not know or none	36	2.8	16.6	7.6–32.4
Owns a home				
No	815	63.0	30.3	27.2–33.5
Yes	478	37.0	37.4	33.2–41.8
Vulnerability condition				
No	1206	93.7	33.2	30.6–35.9
Yes	81	6.3	27.1	18.5–37.8
Planned pregnancy				
No	678	52.4	29.7	26.4–33.3
Yes	615	47.6	36.4	32.7–40.3
Number of living children				
0–1	695	53.8	32.9	29.5–36.5
2 − 3	551	42.7	33.0	29.2–37.0
≥4	45	3.5	31.1	19.3–45.9
Experienced health problems during pregnancy				
No	847	67.1	36.5	33.4–39.9
Yes	416	32.9	27.8	23.7–32.3
Pregnancy classified as high risk				
No	665	52.7	35.1	31.6–38.9
Yes	598	47.3	32.1	28.4–35.9
Attended a childbirth preparation class				
No	713	56.5	29.1	17.0–25.9
Yes	549	43.5	39.7	20.8–35.6
Duration of childbirth and labor				
0–11 h	872	68.1	34.5	31.4–37.7
12–23 h	267	20.9	31.8	26.5–37.6
≥24 h	141	11.0	28.3	21.5–36.3
Type of facility for childbirth care				
Private	804	62.2	30.9	27.8–34.2
Public	488	37.8	36.2	32.1–40.6
The physician attending the childbirth was a gynecologist				
No	157	12.1	41.4	33.9–49.2
Yes	878	67.9	33.3	30.3–36.5
Does not know	259	20.0	26.2	21.2–31.9
The attending physician was a general practitioner				
No	659	50.9	31.1	26.8–33.8
Yes	296	22.9	40.2	34.7–45.8
Does not know	339	26.2	31.8	27.1–37.0
Sex of the professional who attended the childbirth				
Male	605	46.7	36.3	32.6–40.2
Female	653	50.5	30.3	26.9–33.9
Does not know	36	2.8	22.2	11.5–38.5

95% CI = 95% confidence interval. n = number of participants.

The prevalence of humanized childbirth in the sample was 32.9% (95% CI 30.4–35.5). Table 1 displays the prevalence rates for each variable studied, highlighting that the occurrence of humanized childbirth was more frequent among women residing in the city of Cali (43.8%) and those with a higher socioeconomic status (42.8%); women who delivered in their own homes (37. 4%); women whose deliveries were not attended by a gynecologist (41.4%); women whose deliveries were attended by a general practitioner (40.2%); women who attended an antenatal course (39.7%); and women who planned their pregnancies (36.4%).

Table 2 shows the crude and adjusted PRs for the variables considered at each level in the hierarchical conceptual model, with their respective 95% confidence intervals. Among the factors associated with childbirth, the following variables showed an association with childbirth in the crude analysis: place of residence, own home, planned pregnancy, health problems during pregnancy, attendance at an antenatal course, type of facility, sex of the professional attending the childbirth, and whether the childbirth was attended by a general practitioner or a gynecologist. When multicollinearity was assessed using Spearman’s correlation matrix, values higher than 0.45 were not found.

**Table 2:** Crude and adjusted associations to humanized childbirths, Colombia, 2018 (n = 1294).

Variables by hierarchical levels	PR (95%CI)	APR (95%CI)	p (APR)
**Level 1**			
City			
Bogotá	Ref.	Ref.	0.0001
Bucaramanga	0.46 (0.27–0.76)	0.45 (0.27–0.76)	
Cali	2.01 (1.42–2.84)	2.00 (1.41–2.84)	
Manizales	1.92 (1.37–2.69)	1.91 (1.36–2.69)	
Medellín	1.91 (1.37–2.66)	1.91 (1.36–2.67)	
Pasto	1.15 (0.76–1.75)	1.16 (0.75–1.75)	
Marital Status at time of childbirth			
Without a partner	Ref.	Ref.	0.4642
With a partner	1.01(0.84-1.21	1.06(0.89-1.28)	
Age of a childbirth (years)			
14–17 years	Ref.	Ref.	0.9212
18–26 years	1.06(0.78–1.44)	0.94(0.70–1.26)	
27–34 Years	1.11(0.81–1.52)	1.01(0.73–1.35)	
≥35 years	1.10(0.76–1.60)	0.96(0.66–1.38)	
**Level 2**			
`Education			
Elementary	1.06(0.86–1.29)	1.10(0.91–1.35)	0.3347
Secondary	Ref.	Ref.	
Higher education	1.01(0.83–1.22)	1.13(0.92–1.40)	
Socioeconomic stratum			
Under	Ref.	Ref.	0.1315
Medium	0.89(0.74–1.06)	0.82(0.68–1.01)	
High	1.23(0.76–2.09)	1.02(0.62–1.67)	
Employed at the time of childbirth			
No	Ref.	Ref.	0. 8805
Yes	0.98(0.84–1.15)	0.98(0.83–1.17)	
Social security affiliation regime			
Contributory	Ref.	Ref.	0.1118
Subsidized	0.95(0.81–1.11)	0.84(0.70–1.01)	
Special	0.83(0.42–1.65)	0.91(0.43–1.93)	
Does not know or none	0.48(0.23–1.01)	0.48(0.23–1.01)	
Homeownership			
No	Ref.	Ref.	0.4451
Yes	1.23(1.05–1.44)	1.06(0.91–1.23)	
Vulnerability condition			
No	Ref.	Ref.	0.3998
Yes	0.81(0.56–1.17)	0.85(0.60–1.22)	
**Level 3**			
Planned pregnancy			
No	Ref.	Ref.	0.1308
Yes	1.22(1.04–1.42)	1.12(0.96–1.30)	
Number of living children			
0-1	Ref.	Ref.	0.4701
2-3	1.00(0.85–1.17)	1.09(0.94–1.28)	
≥4	0.94(0.60–1.47)	1.09(0.71–1.67)	
Health problem during pregnancy			
No	1.31(1.09–1.56)	1.29(1.07–1.56)	0.0064
Si	Ref.	Ref.	
Pregnancy classified as high risk			
No	1.09(0.93–1.28)	0.98(0.83–1.16)	0.8891
Si	Ref.	Ref.	
Attended a maternity preparation class			
No	Ref.	Ref.	0.0008
Si	1.36(1.16–1.58)	1.30(1.11–1.52)	
**Level 4**			
Childbirth duration (hours)			
0-11 h	1.21(0.92–1.60)	1.24(0.95–1.62)	0.2194
12-23 h	1.12(0.81–1.58)	1.13(0.83–1.54)	
≥24 h	Ref.	Ref.	
Type of childbirth facility			
Private	Ref.	Ref.	0.1367
Public	1.17(1.01–1.36)	1.13(0.96–1.34)	
The professional who attended the childbirth was a gynecologist			
No	Ref.	Ref.	0.0053
Yes	0.80(0.65–0.99)	0.88(0.68–1.13)	
Do not know	0.63(0.48–0.83)	0.48(0.41–0.81)	
The attending physician was a general practitioner			
No	Ref.	Ref.	0.3433
yes	1.33(1.11–1.59)	1.02(0.82–1.27)	
Does not Know	1.05(0.86-1.28)	1.20(0.92-1.55)	
Sex of the professional who attended the childbirth			
Male	Ref.	Ref.	0.0093
Female	0.83(0.71–0.97)	0.81(0.69–0.98)	
Does not know	0.61(0.32–1.13)	0.59(0.32–1.0	

PR: Prevalence Ratio; APR: Adjusted Prevalence Ratio; 95% CI = 95% confidence interval;

p (APR): p value; Ref: reference value.

After multivariate analysis, the following variables were associated with humanized childbirth: city of residence, health problems during pregnancy, attendance at antenatal classes, not knowing whether the childbirth was attended by a gynecologist, and the sex of the professional attending the childbirth. The results of the goodness-of-fit tests of the final multivariate model were adequate. The deviance, chi-square, and the model specification tests showed values of p > 0.05.

## Discussion

This study found that the prevalence of humanized childbirth in six capital cities of Colombia was 32.9% (95% CI 30.4–35.5). This means that only one in three women experienced humanized care during pregnancy, labor and delivery, fulfilling the attributes of humanized care, as outlined in current recommendations [2,3] and Law 2244 of 2022 [11]. The prevalence reported here was lower than those reported in other studies, such as Bante et al. in Ethiopia (38.4%; 95% CI 33.7–42.0) [32], a study contucted in Kenya (64%) [33], another study in Ethiopia (56.3%) [34], a study by Tarekenge (65.8%) [35], a study in Costa Rica (50.5%) [36], and a study in Peru (35%) [37].

Conversely, the prevalence of humanized childbirth in this study was higher than that reported by Pereira et al. (19.2%) [38], a study in Mexico (8.4%) [14], and a study in Chile (20.7%) [39]. In these studies, more offensive treatment was reported in public facilities than in private ones, and the incidence of offensive treatment was higher among younger pregnant women (aged 18–29 years), indigenous women, and those with non-heterosexual sexual orientation. These differences may be attributed to the instrument used and the method by which the prevalence of humanized childbirth was estimated in our study. Specifically, this study used a synthetic index that comprehensively captures the multiple human, technical, and procedural dimensions throughout the continuum of maternal care.

The factors associated with humanized childbirth were as follows: the city of residence of the pregnant woman, the presence of health problems during pregnancy, attendance at antenatal course, the sex of the attending professional, and whether the attending professional was a specialist in gynecology and obstetrics. The differences between cities may be explained by variations in how women interact with the health care team, the health care delivery model, and the quality of maternal services in each city, as well as differences in performance of local health system governance in each city [40]. Further studies are needed to clarify these associations.

The association between humanized care and the absence of health problems during pregnancy probably is explained by the fact that complications often require more urgent interventions, which can limit the support available to women, increase stress among healthcare personnel, and make it more difficult to adhere to humanized care protocols. Women who experience complications may also have higher expectations regarding the quality of care due to their greater awareness of potential risks. A study in Malawi found that women without complications during pregnancy reported the highest rates of humanized childbirth [41].

Attendance at antenatal classes was identified as a protective factor for humanized care. These classes facilitate early connections to humanized care, improving interactions between healthcare providers and women. According to the Ministry of Health and Social Protection of Colombia, antenatal classes help develop essential skills for pregnancy, childbirth, and postpartum care [42]. Evidence shows that antenatal education improves pain control, reduces stress, increases vaginal deliveries, and promotes breastfeeding self-efficacy [43,44].

Other finding was that women attended by female healthcare providers were less likely to experience humanized childbirth. Previous studies in Malawi [41] and Ethiopia [32] showed that deliveries attended by male providers were associated with higher scores for respectful humanized care. A possible explanation is that women may have different expectations of care based on the gender of the provider, often expecting more support from female providers or perceiving male providers as more capable. Studies also suggest that female healthcare workers face gender-based discrimination and inequalities, which could influence their perceived quality of care [45,46]. Further research is needed to explore the role of gender and women’s perceptions of maternal care in Colombia.

Finally, the fact that humanized childbirth was lower among mothers who did not know if the attending professional was a specialist in gynecology and obstetrics indicates that there are aspects to be improved in the communication and interaction between health professionals and the mother. This finding can be explained by the conditions at the time of childbirth, which can create feelings of tension and anxiety among the health professionals and in the women, causing them to overlook this aspect.

This study has several strengths. First, it is one of the first quantitative studies conducted in Colombia on this topic, enabling the estimation of prevalence and associated factors in six capital cities. Second, the use of population-based probability sampling allowed for the selection of a sample size sufficient to identify differences between cities and guarantee the precision of the estimates. Another notable strength is the development, validation, and use of an instrument that included all stages of maternity care, from antenatal care to the puerperium. Lastly, the rigor of the statistical methods used strengthens the internal validity of the study.

However, there are several limitations to consider when generalizing the findings. First, as with any cross-sectional study, it cannot establish a cause–effect relationship. Second, the study only included pregnant women from urban areas, particularly capital cities, which may lead to differences in the quality of care compared to women living in small towns or rural areas. Third, potential selection bias should be considered. To evaluate this, we compared certain sociodemographic and healthcare variables, such as maternal age, maternal education, affiliation to the social security health system, number of children, marital status, and attendance at antenatal checkups, among all vaginal births in urban areas in 2018 of women residing in the six selected cities with the characteristics of the sample based on vital statistics data from DANE. No absolute differences of proportions >13% were found, which could be considered clinically relevant. This provides evidence of the representativeness of the study sample and argues against the presence of selection bias. Similarly, only women who had vaginal live births were included in the analysis of humanized care results. This limitation is significant because the cesarean delivery rate in Colombia reached 46.4% in 2015 and 44.6% in 2020 [47], which means that the results apply only to pregnant women who had vaginal births in urban areas.

Given the complexity of measuring humanized care because of its multidimensional nature, and the absence of a reference standard instrument, it was necessary to develop and validate a tool. This instrument would benefit from comparisons with other questionnaires and evaluations in different settings to estimate its concurrent or discriminant validity.

In this context, and analyzing the data from the present study, we assessed the relationship between the humanized childbirth variable and a question from the questionnaire that broadly estimated satisfaction with the care provided during childbirth: “Would you recommend this facility?” The analysis yielded a PR of 18.3 (95% CI 8.2–40.8), adjusted for all variables in the final model (Table 2). This indicates that women who would recommend the facility were 18.3 times more likely to have experienced humanized childbirth compared to women who would not recommend the facility. This result supports the concurrent validity of the humanized birth measure used in the study.

Additionally, owing to mobility restrictions caused by the COVID-19 pandemic, interviews were conducted by phone. While this can be seen as a strength because it facilitated communication, it may also be a limitation if the selected women did not have access to efficient communication devices or lived in areas with limited access. Another limitation was the inability to obtain reliable information on the income of the pregnant women, as many experienced changes in their income due to the pandemic. Furthermore, the COVID-19 pandemic increased the time between childbirth and the interview, which may have led to recall bias. However, studies have shown that women generally remember their maternity care experiences over time [48,49]. We hope that these limitations did not introduce significant bias. In any case, it would be a nondifferential bias, which would not invalidate the results of the study. Moreover, the fact that the study participants were women with live vaginal births from urban areas may limit the generalizability of the findings.

## Conclusion

The prevalence of humanized childbirth in the sample of participating cities was 32.9% (95% CI 30.4–35.5). The factors associated with humanized childbirth included the place of residence of the pregnant woman, the presence of health problems during the pregnancy, attendance at the antenatal course, the sex of the attending professional, and whether the attending professional was a gynecologist–obstetrician. These findings provide important evidence for decision-makers to adjust maternal and perinatal care at each stage. It is recommended to monitor the compliance and implementation of current legislation. The validated instrument proposed here can be used for future research to ensure that health services become spaces that promote humanized care.

## Supporting information

S1 Appendix Humanized childbirth questionnaire. (DOCX)

S2 Appendix Construction of the index of humanized birth. (DOCX)
