# Supplementary material for: Humanized childbirth in Colombia: Prevalence and associated factors
Source: PLoS One. 2025 Jul 11;20(7):e0326766. doi: 10.1371/journal.pone.0326766 (PMC12250279; doi:10.1371/journal.pone.0326766)
Supplement: S1 Appendix — (DOCX) [file pone.0326766.s001.docx]

**Appendix 1. Humanized childbirth questionnaire**

**Table S1. Stages, domains, and items of the humanized childbirth questionnaire**

|  |
| --- |
| 1. **Prenatal care** |
| **Communication and support** |
| During most of your prenatal care (PNC) appointments, were you informed about your pregnancy status? |
| Were you informed about the possible risks? |
| Were you allowed to attend the PNC accompanied? |
| Did they resolve your doubts? |
| Did they listen to you attentively? |
| **Decent treatment** |
| In general, was the treatment received from the staff of the health institution that attended you during most of your pregnancy checkups discourteous? |
| In general, was the treatment received from the staff of the health institution that attended you during most of your pregnancy checkups aggressive? |
| In general, was the treatment received from the staff of the health institution that attended you in most of the pregnancy prenatal care indifferent? |
| In general, was the treatment received from the staff of the health institution that attended you in most of the pregnancy checkups friendly? |
| **Decent physical environment** |
| Which of the following describes the office where you received the highest number of pregnancy checkups: Privacy |
| Which of the following characteristics describes the office where you received the highest number of pregnancy checkups? Comfort |
| Which of the following describes the office where you received the highest number of pregnancy checkups? Cleanliness |
| 1. **Admission to the obstetrics service** |
| **Decent treatment** |
| When you arrived at the IPS [Institución Prestadora de Salud, Healthcare Provider Institution], were you attended to immediately? |
| How would you rate the information you received from the administrative staff at the time you joined the institution? |
| How would you rate the kind of treatment received from the administrative staff at the time of your admission to the institution? |
| How would you rate the information received from the nursing staff upon admission to the institution? |
| How would you rate the kind treatment received from the nursing staff when you entered the institution? |
| How would you rate the information received from the medical staff upon admission to the institution? |
| How would you rate the kind treatment received from the medical staff when you entered the institution? |
| 1. **Labor** |
| **Professional care and empathy** |
| Did you feel that the healthcare team was aware of your contractions? |
| Did you feel that the healthcare team was concerned about the evolution of your contractions? |
| Did you feel that the healthcare team understood your pain? |
| How would you rate the care you received during labor in terms of the interest shown by the staff in your birthing experience? |
| How would you rate the care received during labor in terms of the attitude of the health personnel (respect, kindness, and listening)? |
| How would you rate the care you received during labor in terms of the information you were given about the progress of your labor? |
| How would you rate the care you received during labor in terms of the explanations provided about what you needed to do to have a successful labor? |
| How would you rate the care received during labor in terms of the support you received during contractions (breathing and relaxation)? |
| How would you rate the care received during labor in terms of the privacy of the facilities? |
| How would you rate the care received during labor in terms of the comfort of the facilities? |
| How would you rate the care received during labor in terms of the treatment by the nursing staff? |
| How would you rate the care received during labor in terms of the treatment by the medical staff? |
| How would you rate the care received during labor in terms of the treatment by other professionals? |
| **Autonomy** |
| During labor, were you allowed to walk? |
| During labor, were you allowed to change body positions? |
| During labor, were you allowed to drink fluids? |
| During labor, were you allowed to listen to music? |
| During labor, were you allowed to make calls? |
| During labor, were you allowed to communicate with anyone? |
| Were you accompanied by a person close to you (family member, spouse, doula, or friend) during the care? |
| **Compassionate pain management** |
| Did you know that you are entitled to receive analgesics during contractions? |
| Were you informed that you were entitled to receive analgesics? |
| Were you offered analgesics? |
| Did you receive analgesics? |
| Were you offered other pain-relief measures? |
| **Procedures not recommended** |
| Were you shaved? |
| Did you have an enema? |
| Did you have an amniotomy? |
| Were you monitored? |
| 1. **Childbirth** |
| **Decent treatment** |
| How would you rate the care you received in terms of interest shown by the staff in your expulsion? |
| How would you rate the care you received in terms of the friendliness of the health personnel during your childbirth? |
| How would you rate the care you received in terms of explanations about what you had to do to facilitate the expulsion? |
| How would you rate the care you received during childbirth in terms of respect for your preferences? |
| How would you rate the care you received during childbirth in terms of respect for your beliefs? |
| How would you rate the care you received during labor and childbirth/cesarean section in terms of the privacy in the childbirth room? |
| How would you rate the care you received during childbirth in terms of the comfort of the room? |
| How would you rate the care you received during childbirth in terms of the treatment received from the nursing staff? |
| How would you rate the care you received during childbirth in terms of the treatment received from the medical staff? |
| How would you rate the care you received during childbirth in terms of treatment by other health professionals? |
| **Nonrecommended procedure-free care** |
| Did you have an episiotomy? |
| Did you have abdominal compression? |
| **Were instruments (forceps) used to help deliver the baby?** |
| **Strengthening the family bond** |
| Were you allowed skin-to-skin contact immediately with your baby? |
| Was the baby placed on your breasts in the childbirth room/surgery? |
| Were you accompanied by a person close to you (family member, spouse, doula, or friend) during the care? |
| 1. **Postpartum** |
| **Counseling and accompaniment** |
| Did you feel accompanied/advised by the nursing staff or other professional to breastfeed your baby? |
| Before leaving the institution, were you provided with healthcare education? |
| Before leaving the institution, were you educated on how to take care of your baby? |
| Was the information given clear? |
| Before leaving the institution, were you given an appointment for a postpartum checkup? |
| How well treated did you feel postpartum? |
| **Comprehensive care** |
| Did you receive a uterine massage after childbirth? |
| Did you have a uterine revision after childbirth? |
| Were you allowed to eat immediately after childbirth? |
| Were you accompanied by a person close to you (family member, spouse, doula, or friend) during the care? |
