# Supplementary material for: Humanized childbirth in Colombia: Prevalence and associated factors
Source: PLoS One. 2025 Jul 11;20(7):e0326766. doi: 10.1371/journal.pone.0326766 (PMC12250279; doi:10.1371/journal.pone.0326766)
Supplement: S2 Appendix — (DOCX) [file pone.0326766.s002.docx]

**Appendix 2. Construction of the index of humanized birth.**

The construction of ISMPH included the following phases:

**Consistency and validity analysis of the humanized childbirth construct**

The questionnaire contained questions that were answered on different scales. Therefore, it was necessary to perform a standardization process to eliminate the scale effect. The min–max method was used [1,2]. The items were collected and converted to an identical range between 0 and 1. The direction of each item was determined according to the nature of the variable: a positive direction indicated a better situation (higher value of the indicator), while a negative direction indicated a worse situation (lower value of the indicator). For instance, a score of 1 indicated humanized childbirth, while a score of 0 indicated nonhumanized childbirth. The following formulas were used:

| Positive indicators:  $I_{x} = \frac{x-\text{Min}_{x}}{\text{Max}_{x}-\text{Min}_{x}}\cdot100$ | Negative indicators:  $I_{x} = \frac{Max_{x}-x}{Max_{x}-Min_{x}}\cdot100$ |
| --- | --- |

$I_{x}$: Item

$x$: Item value

${Min}_{x}$ = Minimum item value

${Max}_{x}$ = Maximum item value

To determine the construct validity of the instrument, separate analyses were performed according to the stages of care evaluated in the form: Prenatal Care; Admission to the Obstetric Service and Preparation for Childbirth; Labor and Childbirth; and Childbirth and Postpartum. First, the internal consistency of the instrument was evaluated using the Kuder–Richarson Coefficient, which showed adequate values for each dimension. Next, a tetrachoric matrix was constructed and a principal component analysis (PCA) was performed. The relevance of the sample for PCA was evaluated using Bartlett’s sphericity tests, which yielded a value of p < 0.05 for all dimensions, and the Kaiser–Meyer–Olkin (KMO) measure of sample adequacy was >0.75, indicating adequate data [3].(table S1)

The relevance of the sample for the PCA was evaluated using Bartlett’s sphericity tests. We obtained a value of p < 0.05 for all the dimensions and considered a value higher than 0.75 for the KMO sample as a criterion of data adequacy [3].

**Table S1. Results of the principal component analysis (total items: 74)**

| **Stages of care** | **Domains** | **# of items** | **Own value** | **% initial variance explained** | **% post-rotation variance explained** | **% accumulated variance** | **Internal consistency**  **KR 20** | **Bartlett’s test** | **KMO** | **Cattell’s scree plot** | **Horn test** | **Velicer test** |
| --- | --- | --- | --- | --- | --- | --- | --- | --- | --- | --- | --- | --- |
| **Prenatal care** | **Communication and support** | **5** | **6.04** | **46.5** | **29.8** | **7.5** | **0.62** | **p < 0.001** | **0.75** | **3** | **3** | **1** |
|  | **Dignified treatment** | **4** | **1.92** | **14.8** | **25.2** |  |  |  |  |  |  |  |
|  | **Dignified physical environment** | **3** | **1.58** | **12.2** | **21.0** |  |  |  |  |  |  |  |
| **Admission to the obstetrical emergency department** | **Dignified treatment** | **7** | **6.08** | **86.8** | **86.8** | **86.8** | **0.92** | **p < 0.001** | **0.81** | **1** | **1** | **1** |
| **Labor** | **Care and empathy from the healthcare team** | **13** | **12.4** | **42.8** | **40.4** | **78.6** | **0.88** | **p < 0.001** | **0.91** | **4** | **5** | **3** |
|  | **Autonomy** | **7** | **5.16** | **17.8** | **18.4** |  |  |  |  |  |  |  |
|  | **Pain management** | **5** | **3.24** | **11.2** | **13.1** |  |  |  |  |  |  |  |
|  | **Care free from nonrecommended procedures** | **4** | **1.94** | **6.7** | **6.9** |  |  |  |  |  |  |  |
| **Birth** | **Dignified treatment** | **10** | **9.73** | **60.8** | **59.5** | **82.4** | **0.88** | **p < 0.001** | **0,92** | **3** | **3** | **1** |
|  | **Care free from nonrecommended procedures** | **3** | **1.94** | **12.2** | **11.6** |  |  |  |  |  |  |  |
|  | **Fostering family bonding** | **3** | **1.5** | **9.4** | **11.3** |  |  |  |  |  |  |  |
| **Postpartum** | **Counseling and support for the mother and baby** | **6** | **4.9** | **49.0** | **44**  **.3** | **64.8** | **0.49** | **p < 0.001** | **0.80** | **2** | **2** | **2** |
|  | **Comprehensive care** | **4** | **1.6** | **15.8** | **20.6** |  |  |  |  |  |  |  |

To decide how many components to retain, the following criteria were used: eigenvalues greater than 1; explained variance greater than 60%; variance explained by the component greater than 10%; and Catell’s scree plot, Horn’s parallel analysis for PCA, and Velicer’s partial correlation test. In addition, to determine the final number of components and items, the research team conducted rounds of analysis. They decided to retain factors based on their theoretical importance within the framework of maternal care in Colombia, in accordance with the results of the statistical tests.

Once the number of components had been selected, the selected components were rotated using the “oblique rotation” method. This was because correlations between items and dimensions of the instrument structure were expected [4]. An absolute value of 0.25 was established as the critical point for item loadings in the components, according to the formula by Norman and Streiner [5]. Finally, the structure of the components was depicted, and internal consistency was estimated for each component, eliminating the items that had loadings >0.25 in more than one factor.

As a result of PCA, the prenatal control dimension consisted of 3 domains and 13 items; the “maternity admission” dimension consisted of 1 domain and 7 items; the “labor” dimension consisted of 4 domains and 29 items; the “childbirth” dimension consisted of 3 domains and 16 items; and the “postpartum” dimension consisted of 2 domains and 10 items. In total, the questionnaire was composed of 74 items, with 12 items from the initial questionnaire being eliminated (Appendix 1).

**Calculation of partial indices (Ip)**

The partial indices in each dimension were estimated by aggregating the scores of their individual indicators or items. Using the weighting method [3], weights were assigned based on the eigenvalues of the components corresponding to each dimension of the PCA construct. The Ip was calculated using the following formula:

$$I_{p}=\sum_{i = 1}^{k} w_{i}\cdot I_{i} Where = \frac{\lambda_{i}}{\sum_{j = 1}^{k} \lambda_{j}}$$

$I_{p}$: Partial index

$w_{i}$: Weight of each component

$I_{i}$ = Indicators or items for each component

$\lambda_{i}$ = eigenvalues of the components

**Calculation of the synthetic index of the experience women have with humanized childbirth (ISMPH)**

To calculate the ISMPH, the research team summed the partial indices. These sums were then weighted according to an ad hoc criterion determined by the research team, considering the importance of each dimension for humanized childbirth. This weighting was took into account the intensity and vulnerability of the labor and childbirth [6,7], as well as unexpected events that might lead to situations in which the mother was under pressure and thus required improved care and behavior from professionals to provide humanized birth care. Accordingly, the following scores were assigned to each of the dimensions: Antenatal Care $w_{1}$1; Admission and Preparation for Childbirth $w_{2}$1.5; Labor and Childbirth $w_{3}$2; Childbirth $w_{4}$2; and Postpartum $w_{5}$1. Finally, the overall humanized birth index was calculated using the following equation:

$$\text{ISPH} = \sum_{i = 1}^{k} w_{i}\cdot I_{i}$$

Where:

ISMPH = Synthetic index to measure women’s experience with humanized childbirth care. $w_{i}$ = Weight or weighting of each dimension, with the condition that

$$\sum_{i = 1}^{k} w_{i} = 1$$

$I_{i} =$Value of the partial indices of each dimension

The synthetic index of the experience women have with humanized childbirth (ISMPH) ranges from 0 to 100, where 0 represents the lowest score in childbirth care and 100 represents the best possible care. Scores of ≥85 were considered indicative of humanized childbirth.

Because there is no history of measuring this construct in Colombia and that the ISMPH distribution did not fit a normal distribution, the research team agreed that an ISMPH score greater than 85 points would be defined as the best experience of humanized childbirth care, which is the main outcome of this study and corresponds to what is hereafter referred to as “humanized childbirth.”

**References**

1. Freudenberg M. Composite indicators of country performance: a critical assessment. OECD Science, Technology and Industry Working Papers. 2003;2003/16. Paris: OECD Publishing. Available from: <https://www.oecd.org/content/dam/oecd/en/publications/reports/2003/11/composite-indicators-of-country-performance_g17a155e/405566708255.pdf>

2. Jacobs R, Smith P, Goddard M. Measuring performance: an examination of composite performance indicators. York (UK): University of York, Centre for Health Economics; 2004. (Technical Paper Series; no. 29). Available from: <https://www.york.ac.uk/che/pdf/tp29.pdf>

3. Nardo M, Saisana M, Saltelli A, Tarantola S, Hoffman A, Giovannini E. Handbook of constructing composite indicators: methodology and user guide. Paris: OECD Publishing; 2008. (OECD Statistics Working Papers; No. 2005/03). Report No.: JRC47008. Available from: <https://publications.jrc.ec.europa.eu/repository/handle/JRC47008>

4. Tabachnick BG, Fidell LS. Using multivariate statistics. 7th ed. Boston: Pearson; 2018. Available from: <http://ndl.ethernet.edu.et/bitstream/123456789/27657/1/Barbara%20G.%20Tabachnick_2013.pdf>

5. Norman GR, Streiner DL. Biostatistics: the bare essentials. 4th ed. Shelton (CT): People’s Medical Publishing House; 2014.

6. Elmir R, Schmied V, Wilkes L, Jackson D. Women's perceptions and experiences of a traumatic birth: a meta-ethnography. J Adv Nurs. 2010;66(10):2142-2153.

7. Hauck Y, Fenwick J, Downie J, Butt J. The influence of childbirth expectations on Western Australian women's perceptions of their birth experience. Midwifery. 2007;23(3):235-47.
